# Equity in out-of-pocket health expenditure: Evidence from a health insurance program reform in Mexico

**DOI:** 10.7189/jogh.13.04134

**Published:** 2023-11-24

**Authors:** Rocío Garcia-Diaz, Sandra G Sosa-Rubí, Rafael Lozano, Edson Serván-Mori

**Affiliations:** 1 Tecnologico de Monterrey, School of Social Science and Government, Monterrey, N.L., México; 2 Center for Health Systems Research, National Institute of Public Health, Cuernavaca, Morelos, Mexico; 3 Institute for Health Metrics and Evaluation, University of Washington, Seattle, USA; 4 School of Medicine, National Autonomous University of Mexico, Mexico City, Mexico

## Abstract

**Background:**

The fragmentation of health systems in low- and middle-income countries (LMICs) deepens health inequities and shifts the economic burden of health care to families via out-of-pocket spending (OOPHE). This problem has been addressed by introducing public health insurance programs for poor people; however, there is a lack of knowledge about how equitable these programs are. We aimed to analyse the long-term effects of the *Seguro Popular* (*SP*) voluntary health insurance program, recently phased out and replaced by the Health Institute for Welfare (*Instituto de Salud para el Bienestar* (*INSABI*)), on OOPHE equity in the poor Mexican population.

**Methods:**

We conducted a pooled cross-sectional analysis using eleven waves of the National Household Income and Expenditure Survey (2002-2020). We identified the effect of *SP* by selecting households without social security (with *SP* or without health insurance (n = 169 766)) and matched them by propensity score to reduce bias in the decision to enrol in *SP*. We estimated horizontal and vertical equity metrics and assessed their evolution across subpopulations.

**Results:**

The program's entry years (2003-2010) show a positive redistributive effect associated with a focalised stage of the program, while oversaturation could have diluted these effects during 2010-2014, with adverse results in terms of vertical equity and re-ranking among insured families. *SP* is more horizontally inequitable than for those uninsured. Within *SP*, the redistributive effect could improve up to 13% if all families with similar expenditures were spending equal OOPHE and horizontal equity was eliminated. Regarding vertical equity, *SP* outperforms the insured population with middle-range coverage some years after the implementation, but this progress disappears.

**Conclusions:**

To achieve universal health coverage, health authorities need to create and execute financial protection mechanisms that effectively address structural inequalities. This involves implementing a more comprehensive risk-pooling mechanism that makes social insurance sustainable in the long-run by increasing the social-economic influx of resources. It is essential to monitor oversaturation and financial sustainability to achieve optimal results. The replacement of the *SP* with *INSABI* highlights the complexity of maintaining a social insurance program where the ideology of different governments can influence the program structure, regulation, financing, and even its existence.

Equity in health financing is a key concern of health systems worldwide, particularly segmented and fragmented ones [1,2]. Health systems must guarantee equitable access to effective and timely health services. However, accessing health services can lead to individuals, mainly those from vulnerable populations, using significant portions of their disposable income to pay privately and compromising their welfare [3,4].

Some studies have analysed equity in all income sources to finance health care reforms in developing and developed countries [5-8]. From all these income sources, out-of-pocket health care expenditure (OOPHE) is recognized as the most unjust, inequitable, and inefficient way to finance health care [9]. Its financial burden is heavier in low- and middle-income countries (LMICs) [10], making its reduction a policy objective in implementing social insurance reforms to remedy regressivity and reduce health care costs for poorer households [11]. The absence of risk pooling mechanisms that limit the consumption of health care by the poor is a critical issue of the segmented and fragmented health system in developing countries [12,13].

Financial risk protection is a key element of universal health coverage (UHC) and the Sustainable Development Goal (SDG) 3.8.2 indicators [14,15]. Social and public insurance schemes, frequently featuring risk-pooling mechanisms, have been proposed by several LMICs as a financing tool to reduce the share of OOPHE [11].

Based on public health evidence, the Mexican Congress approved a reform in 2003 to provide social protection in health to those lacking social security through the System for Social Protection in Health and its financial arm, *Seguro Popular* (*SP*) [16], a public health insurance scheme targeting the poorest uninsured groups. Enrolment in the *SP* was not dependent on health status or pre-existing illness and required no co-payment based on the type of health care received [17,18]. Previous studies have suggested that this program, cancelled in December 2018, has had several positive effects: it contributed to reduced catastrophic and/or impoverishing health spending by its beneficiaries [18], reported greater use and effectiveness of health services [19,20], and exhibited synergistic benefits with other programs such as the conditional cash transfer program (*Progresa-Oportunidades-Prospera* (*POP*)), also cancelled in 2018, in reducing gaps in access to and utilisation of essential health services [21]. Studies have even suggested that it had positive effects on mortality [22,23]. However, a recent literature review highlighted a gap in knowledge regarding the program’s equity effects [24].

We hypothesised that the deterioration in financial protection in the health system for the most vulnerable population in Mexico has led to increased horizontal inequity in health, as measured by unequal OOPHE [25]. Specifically, we expect that OOPHE is unequal in households with a similar ability to pay and that it varies by household socioeconomic status and health financing situation. Thus, improving equity aspects of the health programs could lead to a reduction in redistribution of OOPHE across different households.

For this, we follow the approach suggested by Duclos et al. [26] to inequality decomposition into horizontal inequity, vertical inequity, and re-ranking. The analysis is based on a social welfare function aggregating individual utilities across populations using rank-dependent ethical weights. This provides ethical flexibility to implement sensitivity checks on equity effects in terms of OOPHE that result from *SP* across time and in comparisons with uninsured households and has the advantage of tackling issues of identification of equal households in the expenditure distribution using nonparametric estimation [27,28].

Our study is closest to that of Cavagnero and Bilger [29], who analysed the health financing program reform in Argentina 2001/2002 for all income sources in the health system. Their methodology allows us to assess different aspects of OOPHE for *SP* beneficiaries and households with no insurance. However, there are some differences in the analysis. Instead of covering all sources of health care expenditures, we focus solely on OOPHE associated with a household’s health insurance status. We conduct a long-term equity (horizontal and vertical) analysis of the *SP* program and uninsured populations at different time points, allowing us to examine how equity measures change over time and in relation to *SP* enrolment, highlighting periods of possible oversaturation of the program.

## METHODS

### Settings

We conducted a pooled cross-sectional analysis based on eleven waves of the National Income and Expenditure Household Survey (ENIGH) (2000-2020) [30], a probabilistic, cross-sectional survey implemented biannually since 1992 and administered and managed by the National Institute for Statistics and Geography (*Instituto Nacional de Estadística, Geografía e Informática* (*INEGI*)). It includes a standardised set of income and expense questions and is the most complete source of data of health spending in Mexico.

During 2000-2020, *INEGI*-trained personnel administered face-to-face surveys to 390 313 households, among which we selected households without social security, including those without health insurance (n = 77 496) and those affiliated exclusively to *SP* (n = 97 608). After excluding 3% of households with incomplete data or implausible values in the variables of interest for this study, we obtained an analysis sample of 169 766 households.

### Measures

We began by calculating the total quarterly consumption for each household by adding expenditures on food and beverages, transportation and communication, housing and services, personal care, education, and OOPHE, among others. We defined OOPHE according to the Classification of Individual Consumption by Purpose (COICOP) 2018 [31], including expenditures for medicines and other health products, outpatient care, inpatient care services, and other services (e.g. laboratory analyses and dental services). We also calculated household capacity to pay according to the poverty line defined by Xu’s approach [32]. Expenditure figures are those incurred during the quarter prior to the survey and are expressed in international purchasing power parity dollars (PPP) constant of 2013. Following previous studies, we complemented the OOPHE with expenditure outside the home and both the monetary and non-monetary components of household spending [33].

We classified households according to the two types of members’ medical insurance (*SP* and no insurance affiliation) and created a binary variable accordingly (affiliation to *SP* = one, no health insurance = 0) [17].

We also included the following covariates:

1) Household: age, schooling, employment and marital status of household heads, composition (unipersonal, nuclear, extended or composite), presence of members aged 0-5, ≥55, and ≥65 years, number of equivalent adults, and a factorial index of assets and housing materials as measures of socioeconomic status [34], calculated based on the factor loadings for 2000, where higher values indicated better housing conditions, the participation in a government conditional/non-conditional transfer program.

2) Place of residence: rural/urban (urban ≥2500 inhabitants), access to public services, housing conditions and income according to a social-deprivation index [35] (where higher values indicated more socially developed municipalities), municipality coverage of *SP* (% among population without social security), municipality density ( × 1000 inhabitants without social security) of primary care clinics, inpatient hospital beds (or those in service installed in the hospitalisation area), physicians and dentists, physicians-in-training, and nurses. We obtained the total number of people affiliated with *SP* at the municipal level from the administrative data of *SP* (2003-2019) [36] and the 2020 Mexican Census. We retrieved the total number of people without social security from projections of the National Population Council 1990-2012 [37], the 2015 Intercensal Survey, and the 2020 Mexican Census [38]. We predicted the population without social security at the municipal level for 2013, 2014, 2016-2019 using a linear regression model with robust standard errors for each state. We obtained data on health resources from the Ministry of Health’s Health Care Equipment, Human Resources, and Infrastructure Information Subsystem [39]. Finally, we grouped the 32 Mexican states into seven socioeconomic regions, with one representing the lowest and seven the highest level of development [40].

### Definition of equity point estimates

Using the quarterly consumption distribution (*X*), we obtained the net expenditure distribution (*N*) when discounting OOPHE. Therefore, the relative change in inequality that results from the effects of OOPHE is:

Equation 1) ∆*I* = *I_x_* − *I_N_*

The redistributive change in inequality due to OOPHE identifies the elements of financial protection a social insurance program may have. A positive *ΔI*  means that OOPHE in richer households is higher than in poorer households, causing a reduction in net expenditure distribution, while a negative one suggests OOPHE increases inequality due to higher OOPHE in poorer households. When *ΔI*  identical to zero, either the distribution of OOPHE is equally proportional to the gross expenditure distribution, or families are not spending in health. The redistributive change in inequality due to OOPHE can be decomposed as:

Equation 2) 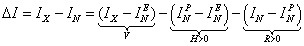


The decomposition results in a series of inequality and concentration indexes (Appendix 1 in the **Online Supplementary Document**). First, vertical equity (*V*) is the decrease in inequality caused by a health system that treats equals equally. To compute it, artificial groups of expenditure equals must be constructed and purged from within group inequality to obtain *I^^E^^__N__*  . *V *is then captured in the difference between *I*_X_ − *I^^E^^__N__*  , which is the progressivity of the OOPHE when horizontal inequity is removed. The second component is horizontal inequity, which measures the increase of inequality due to an unequal net expenditure treatment of households that were initially equal. That is, the difference *I^^E^^__N__*   − *I^^PP^^__N__*   is a consequence of inequality within groups of households equals in terms of expenditure *I^^PP ^^__N__*  . The last component is the re-ranking effect, which identifies households that have changed rankings order in the expenditure distribution due to OOPHE.

### Analysis

We analysed data using Stata, version 17 MP (StataCorp LLC, College Station, Texas, USA). We first described the households’ sociodemographic characteristics and their health expenditures by type of insurance (*SP* and no-*SP*). We then constructed unadjusted linear (for continuous variables) and logistic regression models (for binary variables) to assess independence between groups.

Two aspects were key in analysing the effect of *SP* on OOPHE and its associated equity aspects – accounting for the potential bias of the decision to buy the insurance and identifying equal households in the gross expenditure distribution to assess horizontal inequity in equation 1. In the case of selection bias to buy insurance, we hypothesised that households that were regularly incurring or expecting health expenditures soon were more likely to buy or enrol in a program than those who were not. To assess this source of bias, we used pooled propensity-score matching [41]. We used multiple logistic regression models to estimate the propensity scores (Appendix 2 in the **Online Supplementary Document**) and matched the study sample for all selected covariates [17,42-45]. We use the 1-1 nearest-neighbor algorithm, including caliper = 0.01, non-replacement, and common support. We examined balance in covariates and found balance between comparison groups (Appendix 3 in the **Online Supplementary Document**) [46]. We also found that the matching estimations were insensitive to a hidden bias after computing Mantel Haenszel test (Appendix 4 in the **Online Supplementary Document**) [47,48].

**Figure 1:**
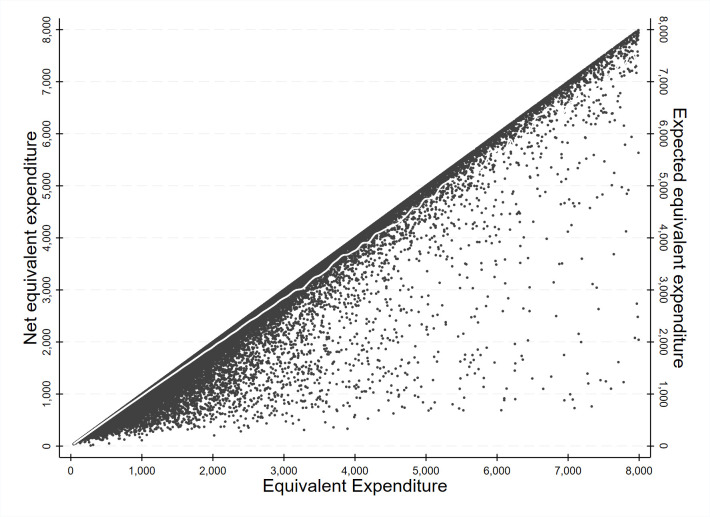
Out-of-pocket health expenditure in Mexico, 2002. Author’s calculations using ENIGH for year 2002. The out-of-pocket health expenditure corresponds to all private health expenditures paid by households in a year.

We performed horizontal and vertical equity analyses among 65 990 matched households. To measure horizontal equity, we used non-parametric estimation to estimate a conditional net expenditure density, allowing us to derive the horizontal inequity measure in Equation 2. We obtained the normative measurement using the inequality indices of gross expenditures, net expenditures, and concentration coefficients of net expenditures according to the ranking of pre-OOPHE expenditures and simulated net expenditures. The analysis is a two-step process, with the first stage being a statistical exercise and the second being the normative measurement. **Figure 1** presents the resulst of the statistical analysis on a pooled sample of ENIGH surveys conducted between 2000 and 2020, showing equivalent expenditure, net equivalent expenditure, and expected equivalent expenditures (the white dots).

## RESULTS

### Descriptive analysis

Households affiliated with *SP* compared to those who have not had slightly older heads of household, with less schooling, greater participation in the labor market, and a higher proportion of men, married, or living in common-law unions (**Table 1**). A higher proportion of these households were nuclear or extended (89.4% vs 81.3%), had children aged 0-5 years (34.8% vs 27.8%), and adults aged ≥65 years (22.8% vs 21.3%). These households also had equivalent number of adults (2.6 vs 2.4), lower socioeconomic level, much higher participation in social programs (50.1% vs 22.6%), residing mostly in rural settings (56.5% vs 35.0%), greater social deprivation, and located in less developed states. Propensity score matching statistically balanced both types of households on all observable covariates (**Table 1**).

**Table 1:** Overall characteristics of analysed households according to *Seguro Popular* and/or *INSABI* affiliation before and after matching process, Mexico 2000-2020, presented as calculated average of percentage (95% confidence interval) unless otherwise specified*

	Before matching		After matching	
	***Seguro Popular*†**	**Without health insurance**	***Seguro Popular*†**	**Without health insurance**
**Number of households, n (%)**	96 496 (56.84)	73 270 (43.16)	32 951 (50.00)	32 951 (50.00)
**Head of household**				
Average age in years	48.88 (48.77, 48.98)††	48.47 (48.35, 48.59)	48.15 (47.98, 48.33)	48.02 (47.84, 48.19)
Female	25.68 (25.41, 25.96)	25.25 (24.93, 25.56)	26.51 (26.03, 26.98)	26.35 (25.87, 26.82)
Schooling				
*None*	13.52 (13.30, 13.74)‡‡	13.97 (13.72, 14.22)	11.73 (11.39, 12.08)	11.85 (11.50, 12.20)
*Elementary*	47.20 (46.89, 47.52)††	42.96 (42.60, 43.32)	41.46 (40.93, 41.99)	41.23 (40.70, 41.76)
*Secondary*	26.82 (26.54, 27.10)††	20.41 (20.12, 20.70)	25.38 (24.91, 25.85)	25.06 (24.60, 25.53)
*High school*	8.16 (7.99, 8.33)††	10.44 (10.22, 10.66)	11.96 (11.61, 12.31)	12.06 (11.71, 12.41)
*College or over*	4.29 (4.17, 4.42)††	12.22 (11.98, 12.45)	9.47 (9.15, 9.78)	9.79 (9.47, 10.11)
Employed in the last month	80.82 (80.57, 81.07)††	78.56 (78.26, 78.86)	79.66 (79.22, 80.09)	80.13 (79.70, 80.56)
Marital status				
*Married/free union*	73.65 (73.37, 73.93)††	65.41 (65.07, 65.76)	65.94 (65.43, 66.45)	65.38 (64.87, 65.89)
*Divorced/separated/widowed*	20.93 (20.68, 21.19)††	24.37 (24.06, 24.68)	24.69 (24.23, 25.16)	24.72 (24.25, 25.18)
*Single*	5.42 (5.28, 5.56)††	10.22 (10.00, 10.44)	9.37 (9.05, 9.68)§§	9.91 (9.58, 10.23)
**Household**				
Composition				
*Unipersonal*	9.82 (9.63, 10.01)††	17.90 (17.63, 18.18)	17.54 (17.13, 17.95)	18.22 (17.80, 18.64)
*Nuclear*	67.07 (66.77, 67.36)††	63.89 (63.54, 64.24)	65.24 (64.72, 65.75)	64.68 (64.17, 65.20)
*Extended*	22.40 (22.13, 22.66)††	17.40 (17.12, 17.67)	16.43 (16.03, 16.83)	16.32 (15.92, 16.71)
*Composite*	0.72 (0.66, 0.77)§§	0.81 (0.75, 0.88)	0.79 (0.69, 0.88)	0.78 (0.68, 0.87)
Any member aged 0-5	34.75 (34.45, 35.05)††	27.83 (27.51, 28.16)	27.32 (26.84, 27.80)	27.00 (26.52, 27.48)
Any member aged ≥55	38.47 (38.16, 38.78)§§	37.85 (37.50, 38.20)	36.42 (35.90, 36.94)	36.25 (35.73, 36.77)
Any member aged ≥65	22.78 (22.52, 23.05)††	21.29 (21.00, 21.59)	19.69 (19.26, 20.12)	19.40 (18.97, 19.82)
Number of equivalent adults, average	2.64 (2.64, 2.65)††	2.41 (2.40, 2.42)	2.37 (2.36, 2.38)	2.36 (2.35, 2.38)
SES index, mean (SD)‡	-0.02 (-0.03, -0.01)††	0.03 (0.02, 0.04)	0.16 (0.15, 0.17)	0.16 (0.14, 0.17)
Beneficiary of any social program§	50.10 (49.78, 50.41)††	22.59 (22.28, 22.89)	25.49 (25.02, 25.96)	25.64 (25.17, 26.11)
**Area of residence**				
Urban	43.52 (43.21, 43.84)††	64.99 (64.65, 65.34)	58.82 (58.29, 59.35)	59.18 (58.65, 59.71)
Social deprivation index, mean (SD)‖	-0.08 (-0.08, -0.07)††	0.10 (0.09, 0.11)	-0.20 (-0.21, -0.19)	-0.19 (-0.20, -0.18)
Coverage of *Seguro Popular*¶	77.43 (77.30, 77.56)††	39.21 (38.95, 39.46)	68.42 (68.18, 68.67)††	67.72 (67.45, 67.99)
Density of primary care clinics**	0.68 (0.68, 0.68)††	0.62 (0.62, 0.62)	0.67 (0.67, 0.68)	0.67 (0.66, 0.67)
Density of inpatient hospital beds**	0.55 (0.55, 0.56)††	0.71 (0.71, 0.72)	0.66 (0.65, 0.67)	0.66 (0.65, 0.67)
Density of physicians and dentists**	1.21 (1.21, 1.22)	1.20 (1.20, 1.21)	1.29 (1.28, 1.31)	1.29 (1.28, 1.30)
Density of physicians-in-training**	0.38 (0.38, 0.39)††	0.44 (0.44, 0.45)	0.44 (0.43, 0.44)	0.44 (0.43, 0.44)
Density of nurses**	2.29 (2.28, 2.31)††	2.19 (2.17, 2.20)	2.51 (2.48, 2.54)	2.50 (2.47, 2.52)
Socioeconomic region				
*Lowest*	14.93 (14.71, 15.16)††	12.99 (12.74, 13.23)	13.25 (12.88, 13.61)	13.63 (13.26, 14.00)
*2*	21.70 (21.44, 21.96)††	18.40 (18.12, 18.68)	17.49 (17.08, 17.90)	17.32 (16.91, 17.73)
*3*	17.12 (16.88, 17.36)††	14.68 (14.43, 14.94)	16.02 (15.63, 16.42)	15.72 (15.33, 16.12)
*4*	25.14 (24.87, 25.41)††	22.82 (22.51, 23.12)	24.94 (24.47, 25.41)	24.91 (24.45, 25.38)
*5*	11.69 (11.49, 11.89)††	13.25 (13.00, 13.50)	14.67 (14.29, 15.06)	14.60 (14.22, 14.98)
*6*	7.72 (7.55, 7.89)††	11.54 (11.31, 11.78)	10.36 (10.03, 10.69)	10.38 (10.05, 10.71)
*Highest*	1.70 (1.62, 1.78)††	6.32 (6.14, 6.50)	3.27 (3.08, 3.46)	3.43 (3.23, 3.63)

CI – confidence interval, SES – socioeconomic status, SD – standard deviation

*Data from the 2000, 2002, 2004, 2006, 2008, 2010, 2012, 2014, 2016, 2018 and 2020 waves of the National Household Income and Expenditure Survey, 2000-2020.

†Beginning 1 January 2020, *Seguro Popular* was dismantled and replaced by *INSABI*, which was charged with ensuring the “free delivery of health services, medicines and other associated supplies” for people without Social Security coverage at all levels of care [49].

‡Factorial index calculated according to the 2000 factor loadings.

§Refers to any government conditional/non-conditional program, particularly he recently cancelled Conditional-Cash-Transfer Program known as *Prospera* (formerly *Progresa or Oportunidades*) (*POP*).

‖Based on the 2000, 2010 and 2020 Population Censuses, as well as data from the 2005 and 2015 Intercensal Surveys [35]. We first made linear predictions for the 2002-2008 and 2012-2018 survey waves. We then estimated a factorial and standardized index based on the factor loadings for 2000.

¶Among population without social security.

^**^× 1000 inhabitants without social security.

††*P* < 0.001.

‡‡*P* < 0.01.

§§*P* < 0.05

Among matched households, differences in quarterly spending and in the financial burden of health care stand out (**Table 2**). Compared to non-*SP* households, those affiliated with *SP* reported lower total quarterly median expenditure ($PPP1972.3 vs $PPP2083.3; *P* < 0.001) and on food ($PPP865.4 vs $PPP893.2; *P* < 0.001), greater reliance on the non-cash component of spending, lower spending on food outside the household, as well as a lower capacity to pay ($PPP1157.3 vs $PPP1276.2; *P* < 0.001) and a higher probability of spending on health (65.6% vs 60.8%; *P* < 0.001). Among households that incurred health spending during the quarter prior to being surveyed, the quarterly OOPEH was similar in *SP* and non-*SP* households ($PPP43); however the weight of the non-monetary component in OOPEH was almost 2-fold among households affiliated with *SP* (24.9% vs 13.7%; *P* < 0.001), while the distribution of OOPEH by component was similar (68% on medicines, 25% on ambulatory care, 2% on hospitalisations, and the rest on other health services or goods). The OOPEH/Total household expenditure and OOPEH/Capacity to pay ratios were also higher among households with *SP* (5.0% vs 4.6%; *P* < 0.001 and 7.8% vs 7.0%; *P* < 0.001 respectively), while the OOPEH/Food expenditure ratio was similar (20%).

**Table 2:** Out-of-pocket health expenditure in households according to *Seguro Popular* affiliation after matching process, Mexico 2000-2020, presented as percentages (95% confidence interval) unless otherwise specified*

	*Seguro Popular*†	Without health insurance
**Number of households (%)**	32 951 (50.00)	32 951 (50.00)
**Total households**		
Total household expenditure, median (IQR)†	1972.29 (1330.33, 2926.12)‡	2083.25 (1349.11, 3335.01)
*Monetary*	70.10 (69.88, 70.32)‡	71.51 (71.29, 71.73)
*Non-monetary*	29.90 (29.68, 30.12)‡	28.49 (28.27, 28.71)
Food expenditure, median (IQR)†	865.38 (578.79, 1264.05)‡	893.22 (584.99, 1347.73)
*Outside the household*	21.67 (21.38, 21.95)‡	24.09 (23.78, 24.39)
*Inside the household*	78.33 (78.05, 78.62)‡	75.91 (75.61, 76.22)
*Monetary*	77.55 (77.27, 77.83)‡	78.29 (78.01, 78.58)
*Non-monetary*	22.45 (22.17, 22.73)‡	21.71 (21.42, 21.99)
Food expenditure/total household expenditure, % (95% CI)	45.28 (45.11, 45.44)‡	43.96 (43.79, 44.14)
Capacity to pay, median (IQR)†	1157.27 (700.19, 1952.47)‡	1276.24 (714.63, 2349.71)
Reported OOP>0	65.58 (65.06, 66.09)‡	60.81 (60.28, 61.33)
**If OOPHE>0**		
OOPHE, median (IQR)†	42.97 (15.32, 114.52)	42.89 (13.92, 118.08)
*Monetary*	75.12 (74.58, 75.65)‡	86.25 (85.81, 86.69)
*Non-monetary*	24.88 (24.35, 25.42)‡	13.75 (13.31, 14.19)
*Medicines and health products*	67.70 (67.24, 68.16)‡	69.05 (68.59, 69.52)
*Outpatient care services*	25.16 (24.76, 25.56)	24.91 (24.49, 25.33)
*Inpatient care services*	2.18 (2.00, 2.35)	2.12 (1.95, 2.30)
*Other health services*	4.97 (4.75, 5.19)‡	3.91 (3.72, 4.11)
OOPHE/total household expenditure	5.04 (4.92, 5.16)‡	4.56 (4.44, 4.67)
OOPHE/food expenditure	19.36 (17.82, 20.89)	18.06 (16.94, 19.19)
OOPHE/capacity to pay	7.85 (7.68, 8.01)‡	7.01 (6.84, 7.17)

SES – socioeconomic status, IQR – interquartile range, OOPHE – out-of-pocket health expenditure

*Data from the 2000, 2002, 2004, 2006, 2008, 2010, 2012, 2014, 2016, 2018 and 2020 waves of the National Household Income and Expenditure Survey, 2000-2020.

†Beginning 1 January 2020, *Seguro Popular* was dismantled and replaced by *INSABI*, which was charged with ensuring the “free delivery of health services, medicines and other associated supplies” for people without Social Security coverage at all levels of care [49].

‡*P* < 0.001.

### Equity analysis

**Table 3** shows the result of Equation 2 decomposition for all the population and different subpopulations according to their health insurance status in all years in the analysis. Each panel shows changes in inequality *ΔI*, followed by the equity estimates on vertical inequity (*V*), horizontal inequity (*H*), and re-ranking (*R*) in all years. The upper panel is the subpopulation without insurance and the lower panel is the subpopulation with *SP*. For all populations, when statistically significant, OOPEH has increased inequality in expenditures. We observed that the years after the implementation of *SP* had more significant increase in changes in inequality when compared to households without insurance (0.0237 for *SP* vs 0.009 for non-insured households in 2006 and 0.005 for *SP* vs 0.003 for non-insured households in 2020).

**Table 3:** Horizontal inequity, vertical equity and reranking of out-of-pocket health expenditure in 2000-2020*

	2000	2002	2004	2006	2008	2010	2012	2014	2016	2018	2020
**Without health insurance**											
*ΔI*	0.001 (0.001)	0.001 (0.001)	-0.0002 (0.001)	0.009 (0.004)§	0.002 (0.001)	0.004 (0.003)	0.008 (0.004)‡	0.005 (0.005)	-0.0004 (0.002)	-0.0007 (0.0009)	0.003 (0.002)‡
*V*	0.004 (0.001)§	0.004 (0.002)§	0.003 (0.001)§	0.013 (0.004)§	0.005 (0.001)‖	0.007 (0.003)‡	0.012 (0.005)§	0.006 (0.005)	0.001 (0.002)	0.001 (0.0008)	0.006 (0.002)‖
*H*	0.0008 (0.0001)‖	0.0006 (0.0001)‖	0.0009 (0.0001	0.0009 (0.0002)‖	0.0005 (0.0001)‖	0.0004 (0.00009)‖	0.0002 (0.0001)	-0.0003 (0.0001)§	0.0005 (0.00009)‖	0.0005 (0.0001)‖	0.0007 (0.0001)‖
*R*	0.002 (0.0003)‖	0.002 (0.0003)‖	0.002 (0.0003)‖	0.002 (0.0008)‖	0.001 (0.0002)‖	0.001 (0.0004)‖	0.003 (0.001)§	0.001 (0.0004)‖	0.001 (0.0003)‖	0.001 (0.0002)‖	0.002 (0.0004)‖
**Sample size**	5432	9507	11 094	2481	3649	3649	934	1658	5011	5155	10 460
*I_X_*	0.557005	0.535393	0.521107	0.474485	0.43640	0.445932	0.492187	0.490332	0.496877	0.458449	0.408730
*I_N_*	0.555264	0.53465	0.519919	0.470889	0.43473	0.442750	0.488335	0.487078	0.495226	0.458271	0.402780
***Seguro Popular*†**											
*ΔI*	-	-	-	0.047 (0.016)‖	0.0237 (0.010)‡	0.003 (0.003)	0.006 (0.004)	0.005 (0.003)	0.003 (0.002)	0.006 (0.002)§	0.005 (0.002)§
*V*	-	-	-	0.056 (0.016)‖	0.028 (0.010)‖	0.006 (0.003)‡	0.007 (0.004)	0.006 (0.003)‡	0.007 (0.002)‖	0.010 (0.002)‖	0.009 (0.002)‖
*H*	-	-	-	0.001 (0.0002)‖	0.0009 (0.0002)‖	0.0005 (0.0001)‖	0.0003 (0.0001)§	0.0004 (0.0001)‖	0.0006 (0.0001)‖	0.0008 (0.0001)‖	0.0007 (0.00009)‖
*R*	-	-	-	0.008 (0.001)‖	0.003 (0.0007)‖	0.002 (0.0005)‖	0.0008 (0.0007)	0.001 (0.0003)‖	0.002 (0.0007)‖	0.003 (0.0005)‖	0.003 (0.0004)‖
**Sample size**	0	0	0	2286	3563	3623	986	1661	4978	5261	10 639
*I_X_*				0.390342	0.392930	0.395214	0.402497	0.402733	0.403366	0.408111	0.377873
*I_N_*				0.364297	0.377335	0.390954	0.400155	0.399982	0.401220	0.405022	0.372723
**Overall**											
*ΔI*	0.003 (0.002)	0.002 (0.001)	0.001 (0.001)	0.005 (0.002)‡	0.001 (0.002)	0.002 (0.001)	0.002 (0.002)	0.007 (0.001)‖	-0.0006 (0.001)	0.001 (0.0006)§	0.002 (0.0009)‖
*V*	0.005 (0.002)§	0.005 (0.001)‖	0.005 (0.001)‖	0.009 (0.002)‖	0.003 (0.002)‡	0.004 (0.001)‖	0.005 (0.002)‡	0.010 (0.001)‖	0.002 (0.001)§	0.004 (0.0007)‖	0.006 (0.001)‖
*H*	0.0007 (0.0001)‖	0.0005 (0.0001)‖	0.001 (0.0001)‖	0.001 (0.00009)‖	0.0006 (0.00007)‖	0.0005 (0.00008)‖	0.0005 (0.0001)‖	0.0008 (0.00008)‖	0.0006 (0.00008)‖	0.0007 (0.00005)‖	0.0007 (0.00007)‖
*R*	0.002 (0.0003)‖	0.002 (0.0004)‖	0.002 (0.0003)‖	0.002 (0.0004)‖	0.001 (0.0002)‖	0.001 (0.0002)‖	0.002 (0.0005)‖	0.002 (0.0002)‖	0.002 (0.0002)‖	0.002 (0.0001)‖	0.002 (0.0002)‖
**Sample size**	5432	9507	11 094	11 622	12 344	12 358	4542	8513	28 637	30 371	35 354
*I_X_*				0.523067	0.475373	0.474204	0.476502	0.442455	0.464380	0.453724	0.435075
*I_N_*				0.519372	0.472864	0.472611	0.474920	0.439769	0.463225	0.452864	0.431441

*Data from the 2000, 2002, 2004, 2006, 2008, 2010, 2012, 2014, 2016, 2018, and 2020 waves of the National Household Income and Expenditure Survey, 2000-2020.

†Beginning with 1 January 2020, *Seguro Popular* was dismantled and replaced by *INSABI*, which was charged with ensuring the “free delivery of health services, medicines and other associated supplies” for people without Social Security coverage at all levels of care [49]. So, the year 2020 refers to *SP* and/or *INSABI*. Bootstrap standard errors are reported in parentheses.

‡*P* < 0.10.

§*P* < 0.05.

‖*P* < 0.01.

The last three rows in each panel of **Table 3** present the decomposition of these changes in inequality across different insurance statuses. *V* is normally referred to in the literature as the main determinant of a change in inequality. We expect vertical inequity to dominate the changes in inequality as it happens in **Table 3**, *V* in relation to both *H* and *R*. *V* measures the progressivity of OPPHE in the expenditure distribution after controlling for OOPHE that treat equals equally. It highlights the possibility that OOPHE may be income based; that is, those households with higher incomes pay more in terms of OOPHE. *SP* is more pro-poor across all years, except from 2010 to 2014. Pro-poor outcomes may be a consequence of the fact that initially, *SP* was targeted to the lowest two deciles in the income distribution [50]. These positive outcomes when compared to uninsured households regarding vertical equity are reversed some years after implementation and when the number of beneficiaries substantially increased during 2009-2011. The accumulated affiliation of the program increased substantially in that period, from 31.1% in 2009 to 51.8% in 2011 and remained in that range until 2019 [51]. We can see those results in 2010, 2012, and 2014, where vertical equity in the non-insured population is higher than for *SP* households. Poorer households with *SP* will pay more for the health services and care they need. The positive vertical equity improved from 2016 onwards.

Horizontal inequity is how OOPHE is redistributed among those households with equal living standards in terms of expenditure levels. In all years, horizontal inequity is higher in *SP* households compared to the non-*SP* ones, highlighting the program’s challenges in targeting resources across similar households. The health expenditures of individuals with almost identical household incomes vary considerably, which results from insufficient health coverage across illnesses and frequent shortages of medicines.

**Figure 2:**
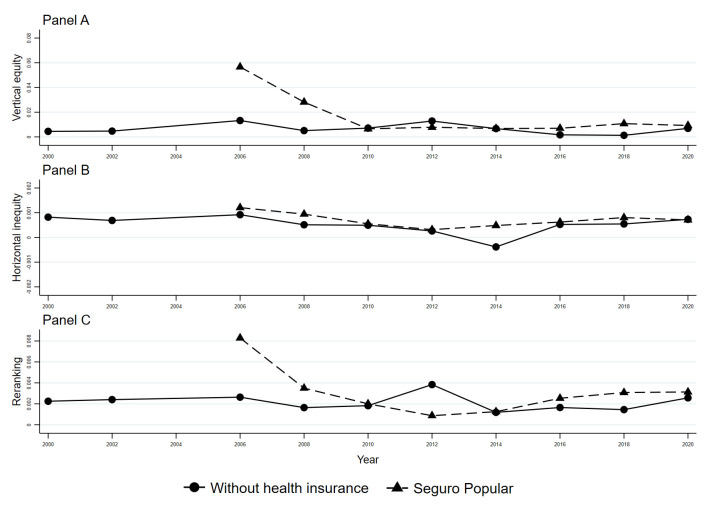
Vertical, horizontal, and reranking equity indicators. **Panel A.** Vertical equity. **Panel B.** Horizontal inequity. **Panel C. **Reranking. Data from the 2000, 2002, 2004, 2006, 2008, 2010, 2012, 2014, 2016, 2018, and 2020 waves of the National Household Income and Expenditure Survey, 2000-2020. The welfare indicator to construct equity indicators is per capita equivalent total expenditure. The equivalence scale we use is the Organization for Economic Cooperation and Development’s equivalent scale [52].

Reranking is the change in inequality after horizontal inequity is removed. In some cases, wealthier households are overtaken by poorer ones after OOPHE. It changes the ranking of families after OOPHE is covered. Reranking in the uninsured subpopulation was the same in all years (*R* = 0.002) and mainly remained the same after the implementation of *SP*. Meanwhile, *SP* beneficiaries faced higher reranking than those uninsured in 2006 (*R* = 0.008) and 2008 (*R* = 0.003). From 2010 to 2014, the reranking was the same or lower, with the best year in 2012 (*R* = 0.0008). In the last stage of the program (2016 to 2020), the reranking was again higher for the *SP* subpopulation. The program's protection in terms of income ranks in the income distribution was limited. We can identify the best period in terms of lower reranking due to OOPHE that happened four years after its implementation. The change in rankings due to OOPHE is related to the lasting effect related to the impoverishing effect in some households in the program since their situations concerning other families with similar resources are profoundly affected after paying OOPHE. **Figure 2** shows all point estimates in three panels showing the equity components for all insurance statuses. In the upper-left panel, we present the horizontal inequity across all years, from 2000 to 2020; in the upper-right, the vertical equity component; and in the lower panel, the reranking effect.

## DISCUSSION

In this long-term study, we found that, as *SP* enrolled more beneficiaries, the redistributive effect of the program diminished, having less or no difference compared to uninsured subpopulations. Its early years show a positive and statistically significant redistributive effect associated with a focalised stage. Our findings align with an analysis using a distributional poverty impact approach. This analysis, conducted in 2006, indicated that the *SP* policy had a more favourable distributional poverty impact for poor households compared to other policy-reducing policies [42]. Between 2010 and 2014, the program did not offer improved financial protection in terms of its redistributive effects than uninsured households, possibly due to oversaturation. This is in line with earlier suggestions it needed to be reassessed and gradually reformed as it aged, and it became increasingly difficult to maintain its benefits observed during 2003-2012 and take the next step toward universal and effective health coverage [18].

Regarding vertical equity, the program was progressive in all years we analysed, with important variations in magnitude, as its early years demonstrated better outcomes for insured compared to uninsured families. However, horizontal inequity was always higher than in uninsured subpopulations, which may indicate *SP* had difficulties in targeting similar health services and resources across its beneficiaries. This forced some households to change their rankings in well-being, measured as per capita equivalent expenditure. Households with access to health services may have access to treatments without full coverage and end up paying a part or all of their costs privately. Our results suggest a social-economic influx of resources or a more comprehensive risk pooling mechanism that makes social insurance sustainable in the long run must be implemented.

There are some differences across insurance types. Government insurance emerges more progressively some years after the implementation, paired with middle-range coverage. In Mexico and other LMICs, government social insurance types are focused only on informal workers or poor, uninsured segments of the population and financed mainly through direct taxes. This certainly limits its progressiveness compared to other countries with social schemes which also include high-income groups [53]. We observed that, in terms of the vertical equity component, the sustained effect, characterised by a significant increase in the number of affiliates, led to the disappearance of progressive effects for insured families compared to uninsured ones.

Horizontal equity in *SP* households is always higher than in uninsured families. Access and coverage were essential determinants for the equity outcomes in our analysis. Both are related to the sustained input of resources to keep an expansion of the programs in terms of coverage paired with the take-up of benefits using access to health care and reduction of OOPHE. The reason people end up spending can be related to insurance coverage and household health characteristics. In a previous analysis [43], we found that older adults or household living in larger cities are better protected against OOPHE health care payments than their uninsured counterparts. Nonetheless, we observed no effect in rural and smaller cities, a result linked to constrained access to limited resources in these regions. There is evidence that some illnesses, such as renal issues, are more likely to induce private payments [54]. This phenomenon is linked to variations in the availability of medicines and coverage across diseases based on insurance alternatives. Hence, there is a need to address changing populations and epidemiological profiles, especially in older subpopulations, as suggested by Parker et al. [55]. However, medicine shortages in some health programmes are widespread, and people must pay either partially or fully for their medicines. A low-cost, limited health service provider is sometimes an effective alternative. For instance, patient’s use of doctor’s offices in private pharmacies in Mexico caused a 5% decline in public outpatient health utilisation [56]. This substitution of public for private health care in outpatient services is not offset by changes in inpatient care, which suggests that the quality of health care in terms of complications leading to hospitalisations is not changing.

In the case of *SP*, the redistributive effect could improve by up to 13% if all families with similar incomes were spending equal OOPHE and horizontal inequity eliminated. An improvement like this could happen if health services included better-designed financial protection on medicines and related health services (such as laboratory testing) when using *SP* health service institutions. However, it is difficult to determine if those inter-household differences can always be considered horizontal equity since we know different households would have different spending patterns. Ultimately, the measure simply highlights the differences for the policymakers to decide how this can be reduced to improve the redistributive effect in the health system performance.

This study has some limitations. First, we did not consider all income sources that would capture the complete financing redistribution by income sources. We also used cross-sectional rather than longitudinal data, which would have allowed us to follow up on life cycle events related to OOPHE among beneficiaries. For instance, evidence suggests that, as individuals get older, they tend to stick to their insurance scheme, especially when household members are diagnosed with chronic illnesses [54,55]. Furthermore, the database we used had no information on the need for health services, and we departed from the average type of households in different categories. Our data offers no information on comorbidities and OOPHE related to certain illnesses. Additionally, we could not examine the total cost of medical expenses when analysing OOPHE. In some cases, individuals may opt for forgoing treatment, increasing long-term deterioration in health, and earning capacity in the future. Third, we still faced recall issues due to the survey structure of ENIGH, as questions regarding OOPHE have a recall period of three months. Fourth, the duration and implementation of *SP* must be considered in interpreting our findings. The program was introduced in 2003 and its rollout participation was gradual across states through local government participation and *POP* program [57-59]. Therefore, our 2004 sample is not nationally representative of *SP*. The program was present in every Mexican state in 2006, was declared universal in 2012 [58], and was replaced by *INSABI* in 2019 [49]. INSABI does not have the structure of a social insurance program where individuals enrol and even make private contributions to their insurance, but instead guarantees health access without individuals enrolling in a program and without making private contributions to their insurance [18]. Lastly, we cannot distinguish between the two programs in the year 2020 in our sample, so we identified them as *SP*/*INSABI*. Nevertheless, we recently found that the dismantling of *SP* in Mexico led to a reduction in health care coverage levels and substantial increases in the proportion of poor households experiencing catastrophic health expenditure and excessive health costs across nearly all Mexican states [25]. These shifts in coverage and financial burdens are key factors in explaining the observed outcomes related to vertical equity and reranking in the years in our study.

## CONCLUSIONS

In a context where a health crisis such as COVID-19 and a risky and profound reorganisation of the Mexican health system converge, the recent cancellation of the *INSABI* program highlights the complexity involved in continuing a financial health protection program and the potential effects that untimely health policies, without taking advantage of previously accumulated knowledge and a solid replacement, could have on the structure of a program, its regulation, its financing and even its existence. In Mexico and other LMICs, achieving a universal health care system requires guaranteeing access to comprehensive and quality services guided by principles of citizenship, without any discrimination whatsoever and regardless of employment status.

## Additional material


Online Supplementary Document
