# Supplementary material for: Equity in out-of-pocket health expenditure: Evidence from a health insurance program reform in Mexico
Source: J Glob Health. 2023 Nov 24;13:04134. doi: 10.7189/jogh.13.04134 (PMC10666565; doi:10.7189/jogh.13.04134)
Supplement: Online Supplementary Document [file jogh-13-04134-s001.pdf]

Appendix 1. **The decomposition of changes in inequality into equity components**

Let $F_{X,N}(\cdot,\cdot)$be the joint conditionally distribution function of gross income (X) and net income (N). Let $p = F_X(\cdot)$be the marginal conditional function for gross incomes and $X(p)$ the quantile function for gross incomes, mathematically defined as $X(p) = inf\{s > 0|F_X(s) \geq p\}$ for $p \in [0,1]$ and the mean gross income is defined as, $\mu_X = \int_0^1 X(p)dp$. The q -quantile function for net incomes, the quartile function and the mean net income is defined analogously.

To characterize different welfare scenarios, we use a social welfare function that aggregates individuals' income utility, $U_e(X(p))$, across populations by using rank-dependent ethical weights, $w(p,v)$. The social welfare function is defined as follows,

$$W_X(\varepsilon, v) = \int_0^1 U_e(X(p))w(p,v) \quad (1)$$

The social welfare function in (1) has two important elements that follow precise specifications. First, the rank-dependent formulation captured in the parametrization of the ethical weights,$w(p,v)$, which according to a popular definition in the literature, is given by,

$$w(p,v) = v(1-p)^{(v-1)}, \quad v \geq 1 \quad (2)$$

This formulation allows individual weights to be normalized to one and obeys the transfer principle from which an equalizing transfer (from a richer person to a poor person) should increase welfare. The parameter v is a parameter of inequality aversion. The larger the value of$v$, the more sensitive is the social decision maker to differences in the ranks of individuals, so the more weight is given to individuals. This rank-dependent formulation forms the basis of our measure of re-ranking in the decomposition analysis. The second element in the social welfare function defined in (1) is the individual income utility function, $U_e(X(p))$, one commonly used form of utility function is,

$$U_e(X) = \begin{Bmatrix} \frac{X^{1-\varepsilon}}{1-\varepsilon}, & \text{when } \varepsilon \neq 1, \\ ln(X), & \text{when } \varepsilon\text{=1.} \end{Bmatrix} \quad (3)$$

which allows the welfare judgment of the policy analyst to be contained in the chosen value of the parameterε. The parameter εdetermines the degree of concavity of the utility function: it becomes more concave as εincreases. An increase in concavity raises the relative importance of low incomes because it causes the marginal utility of income to decline at faster rate.

The social welfare function in (1), combining the ethical weights in (1) and the individual utility of income in (3), displays two parameters that capture aversions to differences in the income rankings of individuals and aversion to riskiness in net incomes. From this dual formulation it is possible a measure of income inequality can be derived as:

$$I_X = \int_0^1 \frac{\mu_X - X(p)}{\mu_X} w(p,v)dp \quad (4)$$

This income inequality function combines Gini and Atkinson's indices of inequality. This serves as a basis for measuring both HI and re-ranking on different normative bases. Using the gross income distribution (X) and net income distribution (N), we can obtain the relative change in inequality that results from the effects of OOP health payments as:

$$\Delta I = I_X - I_N \quad (5)$$

We now proceed to decompose the changes in inequality due to OOP health payments in terms of horizontal inequity (H), vertical equity (V) and re-ranking (R). For that purpose, we require two additional welfare systems and it associated inequality indices. The first welfare system is one in which each individual at rank p in the distribution of gross incomes receives the expected net income of all those at rank$p$,$\bar{N}(p)$, in which social welfare in (1) becomes:

$$W_N^E(\varepsilon, v) = \int_0^1 U_e(\bar{N}(p))w(p,v)dp \quad (6)$$

The second welfare system is one in which, instead of receiving the expected net income, $\bar{N}(p)$, would receive the expected net income utility of those at rank p, in the distribution of gross income, $\bar{U}_e(p)$, then the social welfare function is,

$$W_N^P(\varepsilon, v) = \int_0^1 \bar{U}_e(p) w(p, v) dp \tag{7}$$

From the welfare systems, $W_N^E(\varepsilon, v)$,$W_N^P(\varepsilon, v)$ and $W_N(\varepsilon, v)$, we can derive its associated inequality index as in formula (4). Hence, our redistributive change in inequality can be decomposed as:

$$\Delta I = I_X - I_N = \underbrace{I_X - I_N^E}_{V} - \underbrace{(I_N^P - I_N^E)}_{H>0} - \underbrace{(I_N - I_N^P)}_{R>0}$$

Appendix 2. **Logistic regression model of the association between gender of Seguro Popular[a] affiliation and each covariate[b]**

	Adjusted odds ratio	Robust 95% CI
Head of Household		
Age (in yrs.)	1.00	[1.00, 1.00]
Female	1.38	[1.32, 1.43]
Schooling		
None	2.31	[2.16, 2.47]
Elementary	2.62	[2.49, 2.77]
Secondary	2.56	[2.42, 2.70]
High school	1.72	[1.62, 1.83]
College	Ref.	
Employed in the last month	1.11	[1.07, 1.16]
Marital status		
Married/free union	1.68	[1.58, 1.78]
Divorced/separated/widowed	1.22	[1.16, 1.29]
Single	Ref.	
Composition		
Unipersonal	0.54	[0.46, 0.63]
Nuclear	0.94	[0.81, 1.10]
Extended	1.19	[1.02, 1.39]
Composite	Ref.	
Any member aged 0-5	1.52	[1.47, 1.58]
Any member aged ≥55	1.08	[1.03, 1.13]
Any member aged ≥65	0.80	[0.76, 0.84]
No of equivalent adults	1.07	[1.05, 1.09]
SES index (std)[c]	0.94	[0.92, 0.96]
Beneficiary of any social program	3.45	[3.34, 3.57]
Area of residence		
Urban	0.71	[0.69, 0.73]
Social deprivation index (std)[e]	0.90	[0.88, 0.92]
Coverage of SP[a,c]	4.87	[4.52, 5.24]
Density of primary care clinics[d]	1.48	[1.40, 1.56]
Density of inpatient hospital beds[d]	0.93	[0.90, 0.96]
Density of physicians and dentists[d]	0.90	[0.86, 0.93]
Density of physicians-in-training[d]	0.89	[0.85, 0.93]
Density of nurses[d]	1.02	[1.00, 1.04]
Socioeconomic Region		
Lowest	1.29	[1.17, 1.43]
2	1.79	[1.63, 1.96]
3	1.60	[1.46, 1.74]
4	1.57	[1.44, 1.70]
5	1.35	[1.23, 1.47]
6	1.04	[0.95, 1.14]
Highest	Ref.	
Intercept	0.07	[0.06, 0.08]
Number of households		169,766
McFadden's R^2		0.36
Area under ROC curve		0.82

Notes:

CI: confidence interval; SES: socioeconomic status.

[a]Beginning January 1, 2020, the Seguro Popular Program was dismantled and replaced by the Health Institute for Wellbeing (INSABI, by its initials in Spanish). This Institute was charged with ensuring the “free delivery of health services, medicines and other associated supplies” for people without Social Security coverage at all levels of care.[57]

[b]Data from the 2000, 2002, 2004, 2006, 2008, 2010, 2012, 2014, 2016, 2018 and 2020 waves of the National Household Income and Expenditure Survey, 2000-2020.

[c]Factorial index calculated according to the 2000 factor loadings.

[d]Refers to any government conditional/non-conditional program, in particular, the recently canceled Conditional-Cash-Transfer Program known as Prospera (formerly Progresa or Oportunidades) (POP program).

[e]Based on the 2000, 2010 and 2020 Population Censuses, as well as data from the 2005 and 2015 Intercensal Surveys,[34] we first made linear predictions for the 2002-2008 and 2012-2018 survey waves. We then estimated a factorial and standardized index based on the factor loadings for 2000.

[f]Among population without social security.

[g]x1,000 inhab. without social security.

***$P < 0.001$, **$P < 0.01$, *$P < 0.05$ and $^{+}P < 0.10$

Appendix 3. **Balance of covariates following propensity score matching**

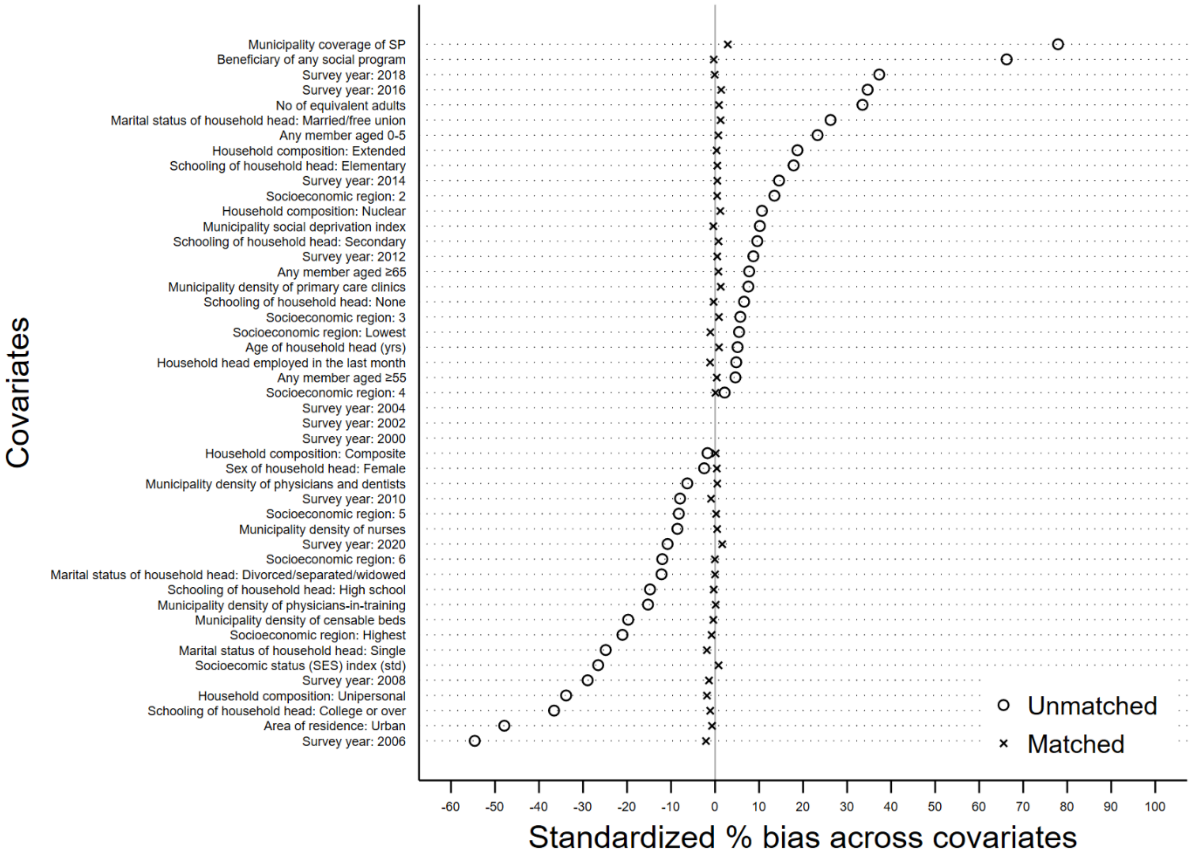

Notes:

Matching was performed using the 1:1 nearest-neighbor algorithm including caliper = 0.01, non-replacement, and common support. Average percentage absolute biases of 19.2% before and 0.8% after matching indicated a balance between comparison groups.

Appendix 4. **Sensitivity analysis: Mantel-Haenszel bounds for affiliation to Seguro Popular**

Notes:

Gamma: odds of differential assignment due to unobserved factors

Q_mh+: Mantel-Haenszel statistic (assumption: overestimation of treatment effect)

Q_mh-: Mantel-Haenszel statistic (assumption: underestimation of treatment effect)

p_mh+: significance level (assumption: overestimation of treatment effect)

p_mh-: significance level (assumption: underestimation of treatment effect)

The Mantel-Haenszel (MH) statistic measured the influence of potential hidden bias on our estimations. This test calculated Rosenbaum bounds for average treatment effects on the treated in the presence of unobserved heterogeneity (hidden bias) between treatment and control cases and provided bound estimates of significance levels at given levels of hidden bias under the assumption of either systematic over- or underestimation of treatment effects. Our results suggest that matching estimations were insensitive to a hidden bias.
